# Interventional Radiology: Towards Personalized Medicine

**DOI:** 10.3390/jpm15110553

**Published:** 2025-11-13

**Authors:** Fabio Corvino

**Affiliations:** 1 Section of Radiology, Department of Biomedicine, Neuroscience and Advanced Diagnostics (BiND), University Hospital “Paolo Giaccone”, 90127 Palermo, Italy; fabio.corvino@unipa.it; Tel.: +39-0817473828; 2 Interventional Radiology Department, AORN “A. Cardarelli”, 80131 Naples, Italy

## 1. Introduction

In recent years, the concept of personalized medicine has moved beyond a theoretical framework to become a tangible clinical imperative. The convergence of advanced imaging, minimally invasive therapies, artificial intelligence, and molecular profiling is rapidly transforming conventional care pathways into highly tailored treatment paradigms. Within this evolving landscape, interventional radiology (IR) stands as a core embodiment of precision medicine, uniquely positioned at the intersection of diagnostic insight and real-time therapeutic action. Unlike traditional specialties, which separate diagnostic evaluation from therapeutic intervention, IR unifies both into a single continuum, diagnosing, characterizing, targeting, and treating disease in real time, with millimetric accuracy and minimal invasiveness [1,2].

This Special Issue of the *Journal of Personalized Medicine* was conceived upon the recognition that radiology is no longer a passive observer in the clinical journey. It is an active driver of clinical decision-making, capable of delivering customized solutions that are patient-specific, organ-specific, and even lesion-specific. The contributions gathered in this Special Issue reflect a transformative shift, from generalized therapeutic approaches to tailored interventions guided by imaging biomarkers, functional anatomy, and real-world clinical needs.

Today, IR stands not as an optional adjunct, but as a central pillar of precision medicine, able to individualize treatment, preserve organ function, reduce systemic toxicity, and offer therapeutic options to patients who previously had none. Together, the articles in this Special Issue demonstrate how minimally invasive, image-guided interventions are reshaping oncologic, musculoskeletal, vascular, and trauma care, consolidating IR as a decisive driver of personalized medicine.

## 2. Emerging Themes from the Special Issue: Interventional Radiology as a Catalyst of Personalized Care

The articles featured in this Special Issue collectively demonstrate how IR is expanding the boundaries of personalized medicine across four strategic domains: oncologic precision therapies, organ-preserving trauma management, patient-tailored vascular and pain interventions, and novel imaging-guided procedures for functional restoration. Together, these studies illustrate a paradigm in which treatment is no longer dictated by disease stage alone, but by the individual biological behavior of the lesion, anatomical variability, functional impact, and patient-specific therapeutic goals.

*Precision Oncology: From Image-Guided Diagnosis to Targeted Therapy.* Several contributions underscore the transformative role of interventional oncology, where treatment is guided not only by tumor morphology, but also by functional status, immune environment, and patient physiology (Contribution 1). Holmium-166 radioembolization and sarcopenia monitoring in hepatocellular carcinoma reveal how body composition metrics, derived from imaging, may serve as dynamic biomarkers to predict early tumor progression, paving the way for IR to act as both a therapeutic and prognostic discipline (Contribution 2). Cryoablation of abdominal wall endometriosis and irreversible electroporation for prostate cancer exemplify true personalization: image-guided technologies that adapt the ablation zone based on tissue type, lesion geometry, and patient functional preservation (Contribution 3). Musculoskeletal tumor interventions highlight a tailored oncologic strategy, choosing between ablation, biopsy, or augmentation based on tumor biology and patient prognosis, demonstrating IR’s role in curative, palliative, and adjunctive settings (Contribution 4).

*Trauma and Emergency Medicine in the Era of Precision.* Historically managed with uniform surgical strategies, trauma care is being revolutionized by image-guided embolization techniques that allow organ preservation, reduction in surgical morbidity, and individualized hemodynamic management. The multicenter Italian experience in splenic artery embolization shows how the personalized selection of embolization techniques (proximal, distal, or combined) results in consistently high splenic salvage regardless of injury grade, reinforcing IR as the cornerstone of non-operative management (Contribution 5). The review on damage control in liver trauma further illustrates how early endovascular control of bleeding, when integrated within hybrid operating environments, enables tailored interventions for hemodynamically unstable patients, offering time-sensitive, patient-specific solutions (Contribution 6).

*Vascular and Functional Interventions for Personalized Quality-of-Life Care.* Beyond survival, personalized medicine also demands the restoration of function and quality of life. IR is uniquely poised to offer minimally invasive, patient-centric interventions. Prostate artery embolization with small-caliber particles highlights individualized device selection based on vascular anatomy and symptom severity (Contribution 7). Genicular artery embolization for osteoarthritis demonstrates how IR can provide durable pain relief and functional improvement in patients unsuitable for surgery, representing a shift from systemic pharmacotherapy to localized image-guided therapy (Contribution 8).

*Image-Guided Functional Targeting and Anatomical Customization.* The inclusion of innovative techniques, such as ultrasound-guided methylene blue nerve localization, reflects the evolution of IR into a discipline that not only treats structural disease but also modulates function with extreme anatomical precision. This represents the next frontier of personalized medicine: functional intervention tailored to individual anatomy, nerve targeting, and specific motor impairments (Contribution 9).

## 3. Looking Ahead: From Personalized to Predictive and Intelligent Medicine

As medicine transitions from a one-size-fits-all paradigm to a tailored, patient-centered approach, IR is poised not merely to participate in this evolution, but to lead it. The studies presented in this Special Issue collectively demonstrate that precision is no longer defined solely by genetic signatures or pharmacogenomics; it is equally embodied in the ability to visually characterize, selectively target, and therapeutically modify disease in real time. This is the essence of IR, a discipline that does not just align with personalized medicine, but actively enables it.

The next transformative leap will be driven by the integration of artificial intelligence and machine learning into the interventional workflow. Radiomics, AI-based decision support, and predictive modeling will allow interventional radiologists to anticipate treatment response, stratify risk with unprecedented accuracy, and dynamically tailor procedures based on patient-specific anatomy, tumor biology, immune status, and body composition. AI-enhanced imaging interpretation and automated segmentation are transforming the interventional suite into a data-driven, intelligent environment, one in which diagnosis and therapy are no longer sequential phases but part of a continuous, adaptive process [3,4].

In this framework, IR becomes the operational engine of precision medicine, transforming imaging from a diagnostic endpoint into a therapeutic roadmap powered by real-time analytics and AI-derived biomarkers. The future of patient care will be image-guided, minimally invasive, data-enriched, and continuously optimized, where every intervention is not only targeted, but *intelligently* tailored to the unique biological and functional profile of each patient [5].

Rather than marking an endpoint, this Special Issue inaugurates a new era in which image-guided therapies, empowered by artificial intelligence, redefine standards of care, improve quality of life, and bridge the gap between diagnosis, prediction, and cure. IR, increasingly supported by data-driven decision tools, stands ready to drive the next great transformation in modern medicine.
